# Revisiting a Previously Validated Temperament Test in Shelter Dogs, Including an Examination of the Use of Fake Model Dogs to Assess Conspecific Sociability

**DOI:** 10.3390/ani9100835

**Published:** 2019-10-20

**Authors:** Shanis Barnard, Danielle Kennedy, Reuben Watson, Paola Valsecchi, Gareth Arnott

**Affiliations:** 1 Center for Animal Welfare Science, Purdue University, 625 Harrison St., West Lafayette, IN 47907, USA; barnard4@purdue.edu; 2 School of Biological Sciences, 19 Chlorine Gardens, Queen’s University Belfast, Belfast BT9 5DL, UK; d30kennedy@gmail.com (D.K.); reuben_watson@outlook.com (R.W.); 3 Dipartimento di Scienze Chimiche, della Vita e della Sostenibilità Ambientale, Università degli Studi di Parma, Parco Area delle Scienze, 11/a, 43124 Parma, Italy; paolamaria.valsecchi@unipr.it

**Keywords:** dog, temperament test, shelter, welfare, validity

## Abstract

**Simple Summary:**

Globally, many unwanted dogs enter rescue shelters. Shelter staff often avail of behavioural tests as an early screening tool to identify areas of concern to minimise the welfare risk associated with long-term kennelling and failed adoptions. A number of requirements need to be verified in order for a test to become a useful assessment tool, including how reliable and accurate the measurements are. For these tools to be widely used, they need to be feasible and reproducible. We refined a previously validated temperament test for shelter dogs’ assessment, developed in Italy, and applied it to two populations of shelter dogs in the UK. The test measured dog behaviour in the kennel, sociability towards people and other dogs, docility to leash, playfulness, cognitive skills, and reactivity. The test proved easy to replicate, with key outcomes that are consistent with existing research on this topic. Furthermore, an additional experiment provided support for the use of fake dogs instead of real ones to assess sociability to dogs. However, we also highlight the importance of interpreting these data with caution, and advocate the use of behavioural tests as a partial screening tool to be used in conjunction with more extensive behavioural and welfare monitoring.

**Abstract:**

This study assessed the feasibility and reproducibility of a previously validated temperament test (TT) for shelter dogs. The test was developed to measure dog behaviour in the kennel, and traits of sociability towards people and other dogs, docility to leash, playfulness, cognitive skills, and reactivity. We introduced the use of differently sized fake dogs to check their appropriateness in correctly assessing sociability to dogs to broaden its applicability (as the original study used real stimulus dogs). We hypothesised that dogs’ responses may be modulated by the body size of the stimulus dog presented. The reduction analysis of the TT scores extracted five main dimensions (explaining 70.8% of variance), with high internal consistency (alpha > 0.65) and being broadly consistent with existing research. Behavioural components that were extracted from the fake dog experiment showed that dogs are likely to show signs of anxiety and fear toward both the real and fake dog. Dogs’ responses towards a real vs. fake stimulus were significantly correlated (*p* < 0.05) and they were not affected by the size of the stimulus (*p* > 0.05). We discuss the importance of interpreting these data with caution and use behavioural tests as a partial screening tool to be used in conjunction with more extensive behavioural and welfare monitoring.

## 1. Introduction

Dogs are a popular choice of pet in United Kingdom (UK) households (nine millions [1]), yet, between 2017–2018, over 56 thousand strays were handled by local authorities in the UK [2]; of these, 24% were passed onto animal welfare organisations for rehoming. There is increasing pressure on shelter staff to rehome dogs and ensure that dog-owner matches are successful, which minimises the rate of returned dogs [3,4,5] due to behaviour problems, particularly aggression, disobedience, and hyperactivity/destruction [3,4]. Behavioural assessments are used within animal shelters to evaluate a dog’s reaction to a range of stimuli and situations to increase the chances of a successful dog-owner match [6,7,8,9]. One of the few comprehensively validated temperament tests for shelter dogs is the one that was developed by Valsecchi and collaborators [9] who have published key test attributes such as reliability and validity [5,10]. Since its publication, the Valsecchi et al. temperament test (hereby TT) has not been, to our knowledge, adopted by other researchers or tested on different shelter populations. A key element of good science, often overlooked in many fields, including behavioural testing and animal welfare science, is the reproducibility of published experimental results [11,12].

In addition to the goodness and appropriateness of the measures used, it is important to also assess test feasibility (i.e., how easily the test can be carried out and the dog behaviour scored, and how practical it is to perform the test in different settings [10]). One of the practical limitations of Valsecchi et al.’s TT was the use of real stimulus dogs to assess intra-specific sociability. The dog-to-dog interaction test is crucial within pre-adoption evaluation to assess a dog’s reaction towards conspecifics. However, the inherent safety and ethical concerns that arise when using real stimuli dogs makes a test less widely applicable: i.e., having to rely on the availability of a calm and well socialised stimulus dog might be challenging in a shelter with fast turnovers. Previous studies have introduced the use of artificial dogs for dog-dog assessments; however, their validity in assessing the sociability to dogs is still debated and in need of further investigation [13,14,15]. Overall, there seems to be a weak correlation between the behaviour of the tested dogs toward the real vs. fake stimulus dogs [13,14]: the inherent stiffness and absence of odour of the fake dog is likely to be among the main reasons causing these different responses. Another confounding factor that has never been taken into account and that could affect the behavioural reaction of dogs during a social interaction is the size of the stimulus [16,17]. The few existing experiments so far have been carried out by using one single artificial dog of medium size [13,14,15]. However, dogs are able to assess and react differently toward conspecifics of different sizes [16,17], an ability termed mutual assessment [18], with size reflecting a proxy of fighting ability, a concept known as resource holding potential (RHP) and observed in many species [18]. Given the above, we introduced, in the TT, the presentation of two fake dogs of different size and compared the reaction of the tested dogs to these fake stimuli and to two real dogs that also differed in size in an analogous way. More specifically, we hypothesise that, consistent with assessment theory, the responses of focal dogs to conspecifics will differ according to the contexts of real/fake and large/small stimulus dog.

In summary, the present study addresses the following objectives:

(1) Adapt and replicate the work by Valsecchi et al. [9] to provide feedback on the replicability and easiness of application of their temperament test in different experimental settings. A supplemented version of the test was implemented in two different rescue shelters in the United Kingdom to do this.
(2) Assess the validity constructs of the test, such as content validity and internal consistency, and compare, where appropriate, the results with those of Valsecchi and collaborators [9]. The results were also used to analyse possible demographic factors (e.g., sex, history, shelter location) that affect the behavioural responses of two populations of dogs to the test.
(3) In the realm of feasibility, introduce the use of differently sized artificial dogs and check their appropriateness in correctly assessing dog-sociability, to both maximise future applicability and minimise ethical/safety concerns that are associated with the use of real stimulus dogs.

## 2. Method

### 2.1. Shelters

A convenient sample of two shelters (in Northern Ireland, UK) was selected for the study (site 1, S1 and site 2, S2). Shelters adhered to local legislation, were registered as no-kill shelters, and had a small team of staff and heavily relied on volunteers and on funding from external sources and charitable donations. Both shelters could care for an average of 45 dogs per week. The dogs at S1 were individually housed within purpose-built kennel blocks and received three 25-min. walks daily. The testing area at S1 consisted of an indoor training room approximately 8 × 11 m with windows and two doors. The dogs at S2, whilst being kennelled indoors at night, were placed in pairs in outdoor runs with chain-linked fencing during the day. The testing area at S2 was in a shed approximately 8 × 10 m, with windows and two doors. Both sites are open to the public for the rehoming of dogs. Tests were performed during closing hours to avoid interruption and distractions.

### 2.2. Subjects

A total of 50 dogs were involved in the study: 20 males (75% neutered) and 30 females (70% spayed) of different breed and mixed breeds. S1 allowed the intake of welfare cases and Staffordshire Bull Terriers and their crosses (5/25), whereas S2 did not. This might, in part, influence any differences found between shelters. Twenty-five dogs were recruited from S1 and 25 dogs from S2. To be eligible for inclusion, dogs had to be declared healthy by staff and undergo veterinary health checks on arrival to the shelter, to be a minimum of 12 months of age and resident at the shelter for at least one week. If the history of the dog was unknown, age was estimated by the shelter veterinarian on the basis of dental and overall health condition. Upon entrance, the dogs were recorded either as previously owned (dogs relinquished by owner for various reasons), welfare case (dog confiscated from the owner), or stray (brought into shelters from the council after short duration in pounds). Dogs that were excluded were those deemed unsafe for testing by shelter staff, due to illness or aggression towards the handlers and dogs that were on any form of medication that could influence behaviour. The mean age of dogs tested was 4.2 years old (SD = ±2.45), with weight 22 kg (SD = ±10.63) and average height of 42.6 cm (SD = ±14.8). Most dogs (62%) were previously owned, 32% were strays and 6% were welfare cases. Reasons as to why the previously owned dogs were relinquished were unknown.

### 2.3. Procedure

The temperament test (TT) that was used was based on the Valsecchi et al. [9] study. The original test consisted of 22 subtests that measured the dog behaviour in the kennel, sociability to humans, intraspecific sociability, docility to leash, playfulness, cognitive skills, and reactivity. We kept the procedures as identical as possible to those that were described in the original paper. An important novel modification of the current study was the addition of two artificial dogs introduced during the dog-dog interaction subtest (described below). Due to the study design, subtests were administrated to all dogs in the same order, with the exception of the real/fake dogs presented in randomised order, as explained later. The experimenters had considerable previous experience in volunteering at shelters and handling dogs; however, they were unfamiliar to the test subjects. One experimenter (female) was always the one administering the test, the other experimenter (male) was observing and scoring the dogs’ reaction.

The test was scored live, although the dog-dog interaction subtest was also recorded for additional detailed behavioural analysis. An initial training phase was conducted before data collection to standardise the protocol (see details in the supplementary material).

#### 2.3.1. Temperament test (TT)

The test protocol and scoring system are detailed in the online supplementary information (Table S1). Initial observations were made from outside the kennel (subtests 1 and 2). The activity of the dog inside its home pen was recorded along with any signs of stereotypical behaviour (e.g., pacing, tail chasing). The experimenter then briefly interacted with the dog first from outside the kennel (subtests 3–5), then from inside the kennel (subtests 6, 7), and took the dog out on a lead into the testing area (subtest 8, 9). In subtest 10, an artificial hand measuring 45 cm in length (Mekuti Assessor Hand) was initially used to pet the dog. If safe, the experimenter would then carry out additional handling (e.g., lift paws, wear muzzle, brush). Subtests 11–13 assessed the dog’s ability to respond to basic commands (“sit”, “stay”) and solve a simple problem task (i.e., retrieve a treat from underneath an upside-down bowl). Playfulness was assessed (subtests 14 and 15) by throwing a ball and a squeaky toy to the dog and then asking it to retrieve. In subtest 16, the fake hand was used to remove a bowl with food from the dog while eating to assess resource guarding. Subtests 17 to 20 were used to assess intraspecific sociability (described below). A startle response (subtest 21) was evaluated by opening an umbrella in the direction of the dog (without contact) and by sounding an air horn. During this reactivity test, habituation to the stimuli was scored and a maximum of ten trials was allowed to habituate the dog if necessary. Finally, the dog was returned to its kennel on a lead (subtest 22).

#### 2.3.2. Dog-Dog Interaction Assessment

This assessment was carried out by the two experimenters: one person holding the stimulus dog (handler 1, H1) and the other person holding the test dog (handler 2, H2). The test was recorded while using a Sony HDR-CX450 camcorder for later analysis, and the dogs’ responses were also rated live by H1 after each encounter following the TT scoring presented in Supplementary Table S1. An artificial large and small dog were used in the assessment (Hansa, UK), and they were chosen for their life-like features (Supplementary Figure S1). The fake stimulus dog breeds selected were a Siberian husky (large-measuring 93 cm in length and 65 cm in height) and a Finnish lapdog (small-measuring 53 cm in length and 37 cm in height).

At site S1, the real large stimulus dogs was a male Staffordshire bull terrier cross (tan four-year-old, neutered, 56 cm in height). The small stimulus dog used was a male Bichon-Frise (white three-year-old, intact, 25 cm in height). At site S2, the real large stimulus dog was a male Alaskan malamute (black and white five-year-old neutered, 66 cm in height), the small real stimulus dog was a male Griffon (black four-year-old, intact, 30 cm in height). The real stimulus dogs were selected from the population within each site and they were described by shelter staff to have a good level of friendliness towards other dogs.

**Figure 1:**
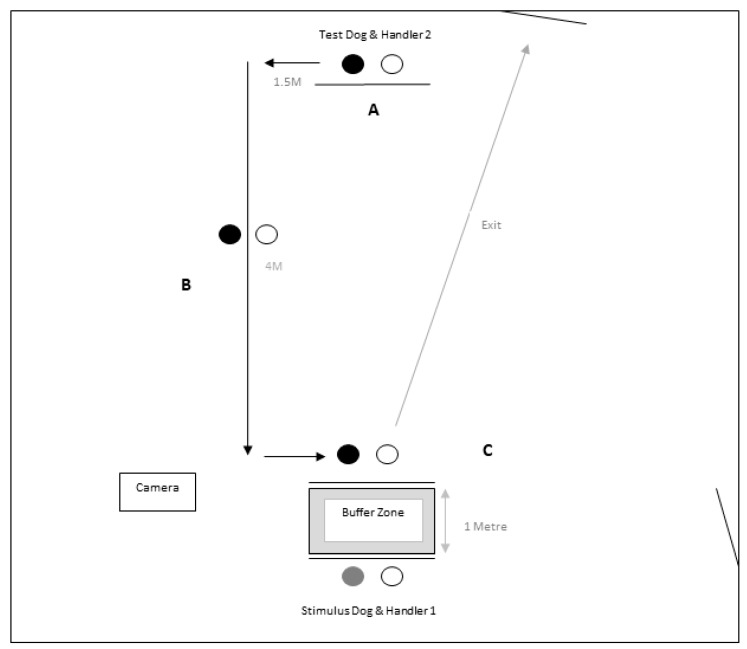
Procedure for dog-dog interaction subtest. Solid circle = handlers, white circle = dogs. Letters indicate the three phases of the protocol: A = distance, B = approach, and C = proximity.

All of the stimulus dogs were provided with regular breaks away from the testing area. Prior to testing, each dog was walked for approximately five minutes to get them used to the handler. A repeated measures design was used to investigate the effect of the independent variable termed ‘stimulus dog’. This consisted of each focal dog (referred to hereafter as the ‘test dog’) being exposed to the four conditions (large real; small real; large fake; and, small fake) in a random order achieved using a random number generator (random.org). Real and artificial stimulus dogs were introduced to the test subjects in the same manner every time. Both real and artificial dogs wore a 1 m lead during all of the testing period. The procedure consisted of three phases:

(1) Distance: H1 and the stimulus dog moved in position 1. H2 entered the room and walked the test dog to the start position (Figure 1A), 5m opposite from H1. H2 remained positioned facing H1 for 15 sec holding the dog on a loose lead.
(2) Approach: H2 followed a predefined route on the floor (drawn using coloured duck-tape) which ended 1 m in front of H1 (Figure 1B).
(3) Proximity: H2 remains in front of H1 for 15 sec. There was a 1 m buffer zone between handlers for safety purposes with no contact between the test dog or fake dog (Figure 1C).

Once this component of the test was complete, the (real) stimulus dog was taken back to its kennels, while the test dog was walked outside the testing area for a few minutes before continuing with the TT.

The test dog was monitored throughout for levels of aggression; however, none of the assessments had to be stopped due to unmanageable aggression.

The behaviour of dogs during this test was scored from video while using an ethogram consisting of 21 behaviours that were compiled from previous work by Shabelansky et al. [14] and De Palma et al. [19] and from scoring a random selection of five videos (Table S2). The behaviours were scored as frequency or duration of behaviour and were analysed while using The Observer XT 14.0 (Noldus, The Netherlands) software.

### 2.4. Statistical Analysis

To enable comparison with Valsecchi et al. [9] the same original scoring was used with some slight amendments. Various scales were used in the scoring process, ranging from two to ten point scales (Table S1). A score of zero was always given when the dog displayed the least desirable behaviour for each subtest (e.g., aggressiveness, not performing the task).

A range of statistical tests were used to assess the validity of measures while using definitions that were provided by Taylor and Mills [10] and Valsecchi et al. [9]. All statistical tests were carried out using IBM SPSS (v.24, IBM, Portsmouth, UK) software with alpha ≤ 0.05.

Content validity: the goodness of chosen measurements i.e., whether an item appears to be measuring the variable that it claims to. Here, for example, different subtests were developed to measure the dog willingness to interact and socialise with humans, so we would expect these items to group into one dimension. A common way to assess content validity in temperament tests is to use a data reduction technique, such as factor analysis or principal component analysis (PCA) [10]. We applied a PCA with varimax rotation and Eigenvalue > 1. Anti-image correlation scores and Kaiser-Meyer-Okin (KMO) for sampling adequacy were checked to ensure that data assumptions were met.

Internal consistency: consistency within components designed to measure the same trait. Here, internal consistency was measured by checking the correlation within each dimension extracted by the PCA while using Cronbach’s alpha test.

Non-parametric tests were then used to assess whether the shelter location, permanence in shelter, sex, neutering status, and homing history affected the PCA component scores. Mann Whitney U test or Kruskall-Wallis tests for two or more independent samples were used for this aspect. Post-hoc pairwise comparisons were conducted where appropriate.

To reduce the number of behavioural variables used to assess the dog-dog interaction, a PCA was run, after data log-transformation, using varimax rotation. Anti-image correlation score and Kaiser-Meyer-Okin (KMO) were checked to ensure sampling adequacy. The principal components scores resulting from this analysis were used as new variables to investigate the effect of the stimulus’ characteristics on the tested dogs’ responses. A Spearman’s rank correlation test was performed to evaluate whether the dogs’ responses to fake versus real stimuli were correlated (matched by size). Subsequently, Wilcoxon signed rank tests were used to assess whether the reaction of the dogs significantly differed when faced with real versus fake dogs or with different sized stimulus dogs.

### 2.5. Ethical Approval

This study received full ethical approval from the participating shelters’ management and the animal research ethics committee at Queens University Belfast (Application approval number: QUB-BS-AREC-16-001). Both of the handlers were experienced kennel workers with good knowledge of the care and handling of dogs in a shelter setting. All the instructions and procedures were agreed in advance of testing. A maximum of 5 dogs was tested each day to avoid undue stress on the real stimulus dogs. The researchers were guided by guidelines laid down by the International Society for Applied Ethology ‘Ethical Treatment of Animals in Applied Animal Behaviour Research’ (ISAE, 2016).

## 3. Results

### 3.1. Temperament Test (TT)

Content validity. The anti-image correlation matrix, presenting the measure of sampling adequacy (MSA) for each variable, showed very poor values for three variables (walking on lead, commands, and attention test; MSA < 0.30). Thus, these were removed from the reduction analysis. The KMO was 0.66 (>0.50), and Bartlett test of Sphericity was significant (*p* < 0.0001) thus meeting the assumption for the analysis to be carried out. The PCA extracted five main factors (Table 1) with the Eigenvalue > 1, explaining 70.8% of variance.

The first component explained the greatest proportion of variance (29.2%) and it had six high positive loadings; these were very specific to human interaction (subtests 3–7 and 10). This first dimension was thus labelled sociability to humans. The second component had four high loadings (subtest 17, 18 19, and 20): approaching large/small fake stimulus dogs and large/small real stimulus dog. This dimension was thus labelled sociability to dogs. The third component had four positive loadings: reactivity (subtest 19), food possessiveness (subtest 16), problem solving (subtest 13), and placing on lead (subtest 8) and was labelled reactivity. The fourth component had one negative loading, observation from distance (subtest 1), and two positive loadings, stereotypical behaviour (subtests 2), and return to kennel (subtest 22). This was labelled adaptation to kennel. The fifth and last component had two positive loadings, squeaky toy and play ball (subtests 14 and 15). This dimension was labelled playfulness.

Internal consistency. Cronbach’s alpha was performed on the three factors with more than two loadings with the same sign. Cronbach’s alpha values were high for all of these factors: sociability to humans = 0.83, sociability to dogs = 0.93, and reactivity = 0.65.

Additional comparisons between dog demographic features and temperament scores. Sex and time of permanence in the shelter did not significantly affect dogs’ behavioural reactions. When analysing whether the dogs’ source had an effect on the factor scores, ‘welfare cases’ (n = 3) were taken out due to their low prevalence and the remaining two categories were compared (relinquished and strays). No significant difference emerged.

**Figure 2:**
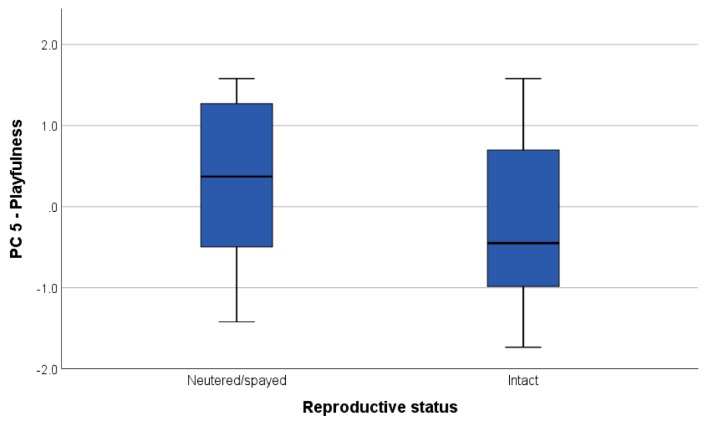
Boxplot showing the significant difference in ‘playfulness’ scores (component 5 of the PCA) between neutered/spayed or intact dogs.

Spayed/neutered animals engaged in significantly more playing (‘playfulness’ factor) than intact dogs (U = 202.5, *p* = 0.039, Figure 2).

**Figure 3:**
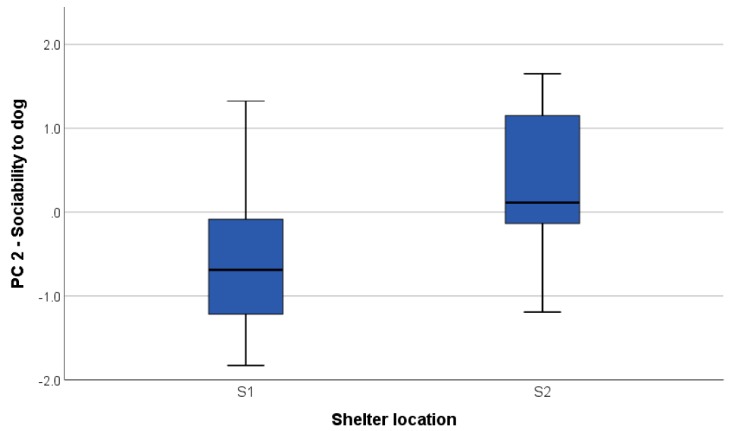
Boxplot showing the significant difference in ‘sociability to dogs’ scores (component 2 of the PCA) between the dogs housed in the two different shelters: site 1 (S1) and site 2 (S2).

There was a significant difference between dogs tested in S1 as compared to S2 in relation to the “sociability to dogs” component (U = 151.0; *p* = 0.002): dogs from S2 were scored higher (i.e., more sociable) that dogs from S1 during conspecific (both real and fake) encounters (Figure 3).

**Figure 4:**
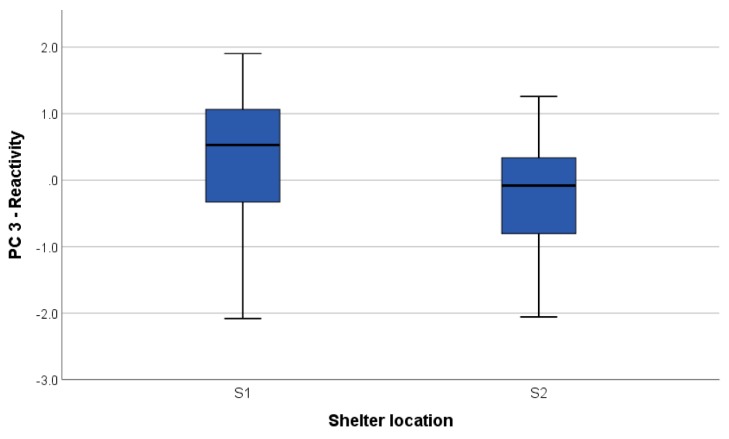
Boxplot showing the difference in the ‘reactivity’ scores (component 3 of the PCA) between the dogs housed in the two different shelters: site 1 (S1) and site 2 (S2). Note: higher scores refer to less reactive dogs and better problem solvers.

A tendency was also found on the 3rd component ‘reactivity’ (H = 216.0; *p* = 0.06). The dogs from S1 were less reactive to sudden stimuli and to food removal and performed better during problem solving than dogs from S2 (Figure 4). No significant difference emerged for the remaining PCA dimensions.

### 3.2. Dog-Dog Interaction

Variables ‘shake off’ and ‘yawn’ were eliminated, as their variance was zero. The anti-image correlation matrix presenting the measure of sampling adequacy (MSA) for each variable showed good values for all remaining variables (>0.40). The KMO was 0.61 (>0.50) and Bartlett test of Sphericity was significant (*p* < 0.0001), thus meeting the assumption for the analysis to be carried out. After visual inspection of the scree plot, four main factors with eigenvalue > 1, were extracted, explaining 45% of variance (Table 2).

The first component explained 16.8% of the variance and was characterised by behaviours typically shown by a more excitable, confident dog, such as pulling toward the stimulus, barking, and displaying play bow. Conversely, ‘investigating the environment’ and ‘looking at handler’ loaded on the same component but with opposite sign. This is more typical of a dog unsure about the stimulus, redirecting its attention toward the handler or the environment. This component was thus labelled confidence. The second component explained 9.9% of variance and the behaviours ‘investigate the stimulus’ and ‘deflection’ loaded higher here. This component was labelled cautiousness. The third component explained 9.3% of variance and it was characterised by higher loadings of the variables ‘lip/nose licking’ and ‘direct staring/stiff posture’. This component was labelled assertiveness. The final component explained 9.1% of variance and it was characterised by dogs showing a very low posture or shrinking back away from the stimulus. This component was thus labelled fearfulness.

Spearman’s rank tests showed that overall the reaction to the fake stimulus dog was significantly correlated to the reaction to the real dog (matched by size, Table 3).

Wilcoxon signed-rank tests were used to investigate further, whether the reaction to dogs’ size (small/large) and nature (real/fake) differed. These paired comparisons showed that there was no significant difference between the groups for any of the comparisons (*p* > 0.05, see Supplementary Table S3 and Figure S2).

## 4. Discussion

The aim of the current study was to replicate and extend the Valsecchi et al. [9] study while using the temperament test (TT) that the authors developed to assess the behavioural profile of shelter dogs. Although several aspects of the original test were validated, no other researchers (to our knowledge) have adopted this test and applied it to different shelter populations, thus a supplemented version of the TT was applied to two populations of dogs housed in two rescue centres in Northern Ireland (UK).

Temperament test. The reduction analysis of the TT scores extracted similar behavioural dimensions to those of Valsecchi et al. [9], especially the first two components ‘sociability to humans’ and ‘sociability to dogs’. This was in line with expectations, as these are the most robust components explaining the majority of variance in both studies (29–18% current work and 21–17% original work). It is interesting to note that, even though the sample size in the current study is smaller, the loadings on the first two components appear to be higher and more defined than in the previous study: in the original work, for example, handling (#10) loaded equally on both component #1 and #2, whereas, here, it only heavily loaded on the ‘sociability to humans’ component. As in the original work [9], the second component ‘sociability to dogs’ is characterised by the four subtests that were designed to assess dog-dog interaction (present study: reaction to real and fake dogs of small and large size; original study: approach and contact with real dogs of same and opposite sex). This result confirms that these scores are highly correlated (internal consistency, α = 0.93) and they appear to be measuring the same behavioural construct [10]. Hence, it can be argued that the reaction of the test dog to a fake dog is more similar to its reaction to a real dog than to any other stimulus presented during the test. A third component that was very similar between the two studies was ‘playfulness’, as described by the two variables ‘play with ball’ and ‘play with squeaky toy’. It appears that playful behaviour (object oriented in this case) can be easily detected with a couple of simple play sessions and that toys elicit a behavioural reaction that is distinct from that shown towards other elements of the test. These first three components, sociability towards people and dogs and playfulness, represent highly desirable behaviours for potential owners that are looking to adopt a dog [20].

The remaining variables loaded somewhat differently here as compared to the original work. First it should be noted that three variables (walking on lead, commands, and attention test) were not included in this analysis, as their MSA (i.e., degree of partial correlation with all the other variables) was not ideal and would have potentially reduced the overall robustness of the test. Interestingly, in the factor analysis by Valsecchi et al. [9], ‘walking on leash’ and ‘commands’ did not load highly on any factor. The response to these subtests may be distinct from the rest of the test as they may depend on the dog’s previous training experience rather than being a mere behavioural predisposition. In a refinement of the test, it could be discussed to drop these scores; however, even though not loading on any factors, these subtests provide important information on any previous training experience as well as the dog’s willingness to engage with the handler (stay close to the handler when on leash, respond when called, focus the attention on the task when asking for a ‘sit’). This information might be valuable when deciding how easy a dog is to train or work with. Differences emerge when considering the other subtests and the result of the reduction analysis of the two studies. In the present work, two components were extracted by the PCA: ‘reactivity’ and ‘adaptation to kennel’. The first one was represented by the variables ‘reactivity’, ‘food possessiveness’, problem solving’, and ‘placing on lead’: indeed, resource guarding can be a form of reactivity to an approaching/sudden stimulus and placing on lead can also trigger a startle/fear reaction similar to that of the reactivity test, especially if the dog is not used to being leashed or if it is afraid of people. The correlation among these elements identifies a calm dog that is able to ignore disturbance while eating, being relaxed to loud noise and to the leash, and also being relaxed enough to focus on the simple cognitive task and motivated to solve it. In the original paper, those variables loaded differently on the extracted factors (Table 4 in Valsecchi et al. [9]). Many factors could account for this difference, i.e., here, we did not include the ‘attention test’ variable in the analysis, hence the redistribution of variables on different components, but more likely these variables may not be ideally designed to measure a separate behavioural construct and they may be underlined by other confounding traits. This is understandable, as leash walking and food possessiveness are again dependent on previous experiences/training and they may not be a proper measure of temperament, but still a useful measure of handling ease and motivation to work.

Finally, the variables on the last component recorded the dog behaviour in their home pen. In the original work, ‘stereotypical behaviour’ loaded on a separate factor and ‘observation from distance’ did not load on any factors, even though the highest loading was on the same factor as ‘stereotypical behaviours’ (Table 4 in Valsecchi et al. [9]). A dog at the front of the kennel (as opposed to the back), returning to its pen without difficulty and not showing stereotypies could indicate a calm temperament, but also a good degree of adaptation to the kennel itself.

Overall, this analysis showed that there is very good overlap between the outcomes of the tests that were carried out by two different research teams on different dog populations, which suggests that the test was well designed to measure temperament traits, such as sociability towards people and other dogs and playfulness. The test appears not to be as robust in assessing other behavioural constructs such as motivation, docility to leash and reactivity. A number of factors may have affected inconsistencies among some of the components that were extracted, including the low variance on some of the variables, the low number of subtests designed to assess one trait, e.g., reactivity (one subtest), problem solving (one subtest), commands (two subtests that imply different underlying skills like ‘come’ or ‘sit’), and the relatively small sample size.

Within the extracted components, the internal consistency was high, especially for the first two components, which further confirmed that items loading on these factors were measuring the same constructs. The alpha value for the third component analysed (‘reactivity’ = 0.65) was indeed lower, supporting the above discussion on this being a less robust component, yet still representing a good correspondence between measures.

The factor scores of the five components extracted were used to explore the effect of dogs’ characteristics and shelter environment on the dogs’ response to the TT. We found that the neutered/spayed dogs scored higher on the ‘playfulness’ component (Figure 2). The effect of spaying and neutering on dog behaviour is not yet clear; some studies investigating the factors affecting aggressive behaviour in dogs, for example, have found weak or no association, often confounded by the fact that, especially in male dogs, aggressiveness is often a cause of castration, rather than a consequence [21,22,23,24]. However, one study on male ferrets found that the incidence of play behaviour was significantly higher after castration [25]. Indeed, more empirical work is needed to explore this topic. Dogs from the two shelters significantly differed on only one component: ‘sociability to dogs’ (Figure 3). This is likely a consequence of the different kennel design, housing system, and management practises characterising the two facilities. In S1, dogs were individually housed with visual barriers between kennels and only interacted with compatible dogs in controlled socialisation sessions. Conversely, dogs in S2 were pair housed in larger outdoor runs for the majority of the time and the wire mesh allowed for them to see the dog in the adjacent kennels. Results showed lower sociability scores for dogs in S1, possibly a consequence of single housing system. It could also be that the population of dogs that the shelters were selecting to intake was different in terms of breed, provenience, and known behavioural problems (e.g., S1 was more likely to accept Staffordshire Bull Terriers and their crosses and welfare cases). However, we could not control for these factors.

Dog-dog interaction. Expanding on previous work [13,14,16], we further investigated whether dogs would modulate their behavioural response when presented with real conspecifics versus fake ones and if the stimulus’ size would affect that response. The PCA extracted four main components. It is interesting to highlight that all of these components include anxiety or stress related behaviour that can be part of different coping strategies that the dogs are showing when faced with the stimulus dog. The first component is described by, on one hand, behaviour typical of a more excitable dog, willing to engage and interact with the other dog; however, behaviours like barking and play bow also loaded here, which can be an indicator of excitement as well as frustration [26]. On the other hand, ‘investigate the environment’ and ‘look at handler’ loaded on this first component (with opposite sign) and these are more clear signs of redirected attention, trying to avoid the interaction with the stimulus and looking at the handler for support [27]. The second component was described by the variables ‘investigate the stimulus’ and ‘deflection’. Dogs investigating the stimulus normally did so in a cautious manner when in contact and they were normally the dogs avoiding looking at the stimulus from far but at the same time breaking eye contact as an appeasing behaviour. These dogs, although somewhat engaging with the stimuli, showed signs of mild fear. The third component was characterised by dogs staring and stiffening at the sight of the stimulus dogs, yet this posture was also associated with lip/nose licking, which suggested that, probably, a component of anxiety was associated with the emotional state of the dogs when facing the stimuli [28]. Finally, behaviours that are typical of a fearful reaction, such as assuming a low body posture and increasing the distance from the stimulus, loaded together on the last factor. These characterise dogs that are clearly avoiding the interaction with a conspecific and they are in a fearful emotional state.

When analysing whether the behavioural component scores that are described above varied in relation to the stimulus presented, we found that the reaction to real versus fake dogs was highly positively correlated and we did not detect any significant difference in the dogs’ responses to the different stimuli despite size (Figure S2). It appears that, in these populations, the fake dog was triggering the same type of behavioural response/coping strategy as the real counterpart, and with no effect of the stimulus’ size. This is in contrast to previous work [16,17] that demonstrated the ability for accurate conspecific assessment in dogs. The conflicting findings likely reflect important contextual differences, with the current study involving stimulus dogs being selected to have a calm, non-aggressive temperament, and the setup lacking vocal cues (e.g., growling) that were a key component, likely used for size assessment in the previous studies [16,17]. The subjects were recruited according to the availability at the time of the study due to the source of dogs (i.e., rescue shelters). It was not possible to select test dogs with a specific body size that matched (or differed from) the stimulus dog size. Future studies should attempt to balance the sample to have a balanced sample of test dogs that are equal, smaller, and larger than the stimulus dog and add the test dog size as a factor, which might have an effect on the dogs’ behavioural response to the subtest.

The use of behavioural tests in shelters have recently been criticised [29]. Behaviour evaluations are said to provide false positives and false negatives, which may hinder the chance of adoption for some dogs [30,31]. It is understood that no test will ever be able to 100% predict behaviour post-adoption and provide new owners absolute certainty that a dog will behave in a certain way, nor should any test claim to be able to do so. The familiar world outside the shelter environment is full of complex situations (i.e., many people in the same household, children, elderly people, other animals) and dogs need some months to adapt to it and show their true personality. The use of standard behavioural tests as screening tools is still important to identify subjects that may need more training, or other targeted interventions (e.g., desensitisation) before adoption and maximise the chances for a successful adoption. These screening tools are also useful for shelter staff to assess and address any behavioural concerns that may affect the dog’s ability to cope in the kennel environment, e.g., very fearful/reactive dogs may be difficult to handle and struggle to adapt, hence impacting on their overall welfare.

## 5. Conclusions

This study focused on an often-overlooked aspect in science: reproducibility of results. A previously validated temperament test for shelter dogs’ assessment, as developed in Italy [9], was applied to two populations of shelter dogs in the UK. We did not expect to fully replicate results from the original work, as we purposefully adapted some parts of the test to be more feasible and widely applicable (i.e., introducing fake dogs as a measure of intra-specific sociability). Nevertheless, the results showed that there was a very good level of overlap between the outcomes of the tests that were conducted in the two different experiments when analysing traits, such as sociability towards people and other dogs and playfulness. This suggests that the test was well designed to measure these behavioural constructs. Other behavioural dimensions that were extracted were less consistent between studies (e.g., motivation and reactivity), likely due to other factors influencing these traits such as training and previous experience, differences in the statistical analysis or other limitations in the test design. Based on the concept of RHP, we explored, for the first time, dogs’ reaction to fake dogs of different sizes and compared it to real dogs matched by size. The results suggest that there is a high correlation between the response to a fake vs. real dog and that we were not able to detect any effect of size on these responses. As a note of caution, we do not recommend using temperament tests and devices (such as a fake dog) in isolation as the only assessment or diagnostic tool for behavioural problems in dogs. However, tests using proxy measures of behavioural assessment can be useful initial screening tools, which have been proven to have a level of validity, ensuring the safety of the handler and contributing to the assessment of behavioural problems that may become a welfare concern in a kennel environment or may be a cause of failed adoption.

## Figures and Tables

**Table 1:** Dimensions extracted by the principal component analysis (PCA). Loadings higher than 0.50 are in bold.

Variables (Subtest #)	Sociability to Humans	Sociability to Dogs	Reactivity	Adaptation to Kennel	Playfulness
Stroking through kennel (#5)	**0.816**	−0.168	0.139	−0.128	0.165
Approach kennel (#3)	**0.805**	−0.051	0.364	0.042	0.068
Side on crouch (#4)	**0.792**	−0.131	0.324	0.157	0.128
Physical contact (#7)	**0.788**	−0.249	0.234	0.133	0.164
Handling (#10)	**0.786**	0.079	0.031	0.091	0.077
Entering kennel (#6)	**0.687**	−0.284	0.054	−0.408	0.069
Approaching large fake dog (#19)	−0.054	**0.944**	−0.034	−0.025	0.023
Approaching small fake dog (#20)	−0.168	**0.913**	0.092	−0.030	0.107
Approaching large real dog (#17)	−0.027	**0.891**	−0.078	−0.117	−0.098
Approaching small real dog (#18)	−0.161	**0.791**	−0.008	0.059	0.301
Reactivity (#21)	0.138	0.038	**0.788**	−0.124	−0.105
Food possessiveness (#16)	0.170	0.029	**0.688**	0.136	0.173
Problem solving (#13)	0.332	−0.087	**0.657**	−0.312	−0.205
Placing on lead (#8)	0.192	−0.069	**0.532**	0.419	0.346
Stereotypical behaviour (#2)	0.476	0.206	−0.092	**0.628**	−0.124
Observation from distance (#1)	0.337	0.049	−0.027	**−** **0.628**	0.221
Return to kennel (#22)	0.168	−0.337	−0.033	**0.526**	0.048
Play ball (#14)	0.240	0.043	−0.113	−0.022	**0.837**
Play squeaky toy (#15)	0.163	0.308	0.231	−0.269	**0.627**
Explained variance (%)	29.23	18.27	8.89	8.26	6.10

**Table 2:** Dimensions extracted by the PCA for the dog-dog interaction test behavioural scores. Loadings higher than 0.50 are in bold.

Behavioural Variables	Confidence	Cautiousness	Assertiveness	Fearfulness
Investigate environment	**−0.772**	−0.075	−0.251	0.010
Pull towards	**0.723**	−0.236	0.076	−0.008
Barking	**0.633**	−0.086	−0.500	−0.014
Look handler	**−0.628**	−0.055	0.015	0.009
Play bow	**0.574**	−0.078	−0.001	−0.065
Investigate stimulus	0.084	**0.847**	−0.058	0.002
Deflection	−0.216	**0.671**	−0.295	0.077
Wagging tail	0.308	−0.475	−0.095	−0.030
Pulling away	−0.158	−0.392	−0.181	−0.138
Lip/nose licking	0.153	−0.060	**0.682**	−0.029
Direct staring/stiff posture	0.501	0.033	**0.548**	−0.087
Lie down	−0.045	0.006	0.456	0.009
Jumping	0.304	0.001	−0.337	0.118
Shrinking back	−0.061	0.107	−0.168	**0.852**
Very low posture/tucked tail	−0.094	−0.105	0.017	**0.605**
Paw lift	0.014	0.164	0.048	**0.577**
Jump on handler	−0.240	−0.068	0.063	−0.247

**Table 3:** Spearman’s rank correlation test comparing dogs’ reactions to real versus fake dog.

Component	Large Real vs. Large Fake	Small Real vs. Small Fake
Confidence	Rho = 0.59, *p* < 0.0001	Rho= 0.56, *p* < 0.0001
Cautiousness	Rho = 0.59, *p* < 0.0001	Rho= 0.37, *p* = 0.015
Assertiveness	Rho = 0.58, *p* < 0.0001	Rho= 0.52, *p* < 0.0001
Fearfulness	Rho = 0.30, *p* = 0.048	Rho= 0.48, *p* = 0.001

## Supplementary Materials

The following are available online at https://www.mdpi.com/2076-2615/9/10/835/s1, Table S1: Temperament test description and scoring system, Table S2: Ethogram used to score the behaviour of the tested dogs when presented with either a real or a fake dog opponent, Table S3: Statistical test and *p*-values used to investigate whether the PC scores from the dog-dog interaction test differed in relation to the stimulus dogs’ size (small/large) and nature (real/fake), Figure S1: Pictures of the large and small fake dogs used for testing, Figure S2: Boxplot showing group comparisons for (**a**) the first PCA component scores ‘confidence’ and (**b**) the second PCA component scores ‘cautiousness’ (**c**) the third component scores ‘assertiveness’ and (**d**) the final component score ‘fearfulness’ in response to the different stimulus dogs’ characteristics (real vs. fake and small vs. large). Dots represent outliers.
