# Supplementary material for: Revisiting a Previously Validated Temperament Test in Shelter Dogs, Including an Examination of the Use of Fake Model Dogs to Assess Conspecific Sociability
Source: Animals (Basel). 2019 Oct 20;9(10):835. doi: 10.3390/ani9100835 (PMC6826718; doi:10.3390/ani9100835)
Supplement: Supplementary file 1 [file animals-09-00835-s001.pdf]

## Supplementary Material

## Procedure

Temperament Test training: During this initial training phase two experimenters (authors DK and RW) were trained by the first author (SB), involved in the original study [9], to perform the test and score the dogs' behaviour. The training was conducted on five dogs and video-recorded in order to conduct independent scoring. At the end of the scoring, the research team examined the results and if there was disagreement on a dog score, a group discussion in front of the video was carried out until a consistent agreement was reached. The five dogs used in the training phase were not included in the final sample.

**Supplementary Table S1** Temperament test description and scoring system.

| Subtest Label | Tester Behaviour | Scoring Description | Score |
|---|---|---|---|
| 1 Observation from distance | Out of the dog's sight, standing at a distance, recording the dog's behaviour (30 s) | Front of kennel | 3 |
| | | Centre of kennel | 2 |
| | | Back of kennel | 1 |
| | | Dog is out of sight | 0 |
| 2 Stereotypical behaviour | Record the presence of stereotypies (pacing, circling, jumping etc.) (30 s) | No | 1 |
| | | Yes | 0 |
| 3 Kennel approach | Approach kennel in a neutral posture, no eye contact, and stop in front of the fence facing sideways. Body posture recorded- friendly (tail wagging, non aggressive-barking, calm etc.), neutral (dog is still, neither threatening or friendly behaviour), fearful (crouched posture, low tail, shaking, whimpering etc.), threatening (barking, growling, lunging towards observer etc.) | Friendly- approach observer for contact | 9 |
| | | Friendly- but not asking for contact | 8 |
| | | Approach with excitement, jumps/struggle to calm down | 7 |
| | | Neutral, calm approach | 6 |
| | | Neutral and still, looking | 5 |
| | | Neutral but avoiding contact | 4 |
| | | Fearful, approach with low posture | 3 |
| | | Fearful, still in centre of kennel | 2 |
| | | Fearful, avoiding, moving away, hiding etc. | 1 |
| | | Aggressive/threatening | 0 |
| 4 Side crouch | Crouch down, side on, near the fence (30 s), observer talks calmly to the dog | Friendly and confident | 3 |
| | | Neutral, less confident | 2 |
| | | Fearful | 1 |
| | | Aggressive/threatening | 0 |
| 5 Stroking through the fence | Call the dog, talk gently, attempt to stroke dog through fence (30 s) | Friendly and confident | 3 |
| | | Neutral, less confident, barking | 2 |
| | | Fearful | 1 |
| | | Aggressive/threatening | 0 |
| 6 Entering kennel | Observer walks into kennel, closes the door. Observer stands still, arms at side, ignoring the dog etc. (30 s) | Dog approaches tester | 2 |
| | | Dog stands still | 1 |
| | | Dog goes away | 0 |
| 7 Physical contact | Bending slightly forward, calling the dog in a relaxed manner, wanting the dog to make contact (30 s) | Friendly- approach observer for contact | 9 |
| | | Friendly- still, not asking for contact | 8 |
| | | Approach with excitement | 7 |
| | | Neutral, calm approach | 6 |
| | | Neutral and still, looking | 5 |
| | | Neutral and avoiding contact | 4 |

| | | | | |
|---|---|---|---|---|
| | | | Fearful, approach with low posture | 3 |
| | | | Fearful, still in centre of kennel | 2 |
| | | | Fearful, avoiding, moving away, hiding etc. | 1 |
| | | | Aggressive/threatening | 0 |
| 8 | Placing on lead | Standing next to the dog, showing the lead (30 s) Dog is allowed to approach, smell and look at it. Record how easy the process is. | Dog is confident and lead is easily put on | 2 |
| | | | Dog is excited/ unmanageable/reluctant to put lead on | 1 |
| | | | Reacts aggressively | 0 |
| 9 | Walking on lead | Walk dog from kennel, down corridor toward testing area. (30 – 60 se) | Easy to handle | 2 |
| | | | Scared, wants to retreat | 1 |
| | | | Unmanageable, pulls | 0 |
| 10 | Handling | Dog is let off lead in test area, free to explore (2 min). Dog is put back on the lead then certain steps are carried out (15 s per step) and for each step the dog reaction was evaluated: | | |
| | | 1. General handling, stroke dog head to tail and try touch and lift front paw | Calm/confident | 2 |
| | | | Freeze, crouched posture, mouth licking but not aggressive | 1 |
| | | | Growling/aggressive | 0 |
| | | 2. Brush head and sides of body | Calm/confident | 2 |
| | | | Freeze, crouched posture, mouth licking but not aggressive | 1 |
| | | | Growling/aggressive | 0 |
| | | 3. Apply muzzle for a few seconds | Calm/confident | 2 |
| | | | Freeze, crouched posture, mouth licking but not aggressive | 1 |
| | | | Growling/aggressive | 0 |
| 11 | Attention testing | Dog is on a lead, observer stands in front of the dog while holding a treat, closes hand while holding treat, holds hand up to the chest. The dog should follow the hand movement and hold a gaze (3 s) | Holds gaze for 3 seconds | 1 |
| | | | Jumps at hand or looks away | 0 |
| 12 | Command testing | a. Come = Off lead, observer stands at a distance and calmly calls the dog to come. | Dog goes to tester | 1 |
| | | | Dog ignores | 0 |
| | | b. Sit = observer shows the dog a treat, moving the treat above the dogs head giving it time to sit (30 s) | Dog sits | 1 |
| | | | Dog ignores | 0 |
| 13 | Problem solving | Off lead, showing the dog a treat and evaluating the interest in the food reward. Showing a second treat and making sure the dog is watching, the treat is placed in an upside down bowl. Attempt to retrieve the treat (30 s) | Accomplish task | 3 |
| | | | Attempts to accomplish | 2 |
| | | | Interested but looks to tester | 1 |
| | | | Not interested in food or task | 0 |
| 14 | Squeaky toy | Off lead, showing dog squeaky toy and throw towards the dog | Dog plays with toy | 3 |
| | | | Dog approaches cautiously | 2 |
| | | | Dog not interested | 1 |
| | | | Dog is scared | 0 |
| 15 | Ball toy | a. Off lead, showing the dog a ball, allowing dog to sniff the ball, throws the ball | Plays with the ball | 1 |
| | | | Dog does not play with the ball | 0 |
| | | b. If the dog brings the ball back, then throw again once more | Was the ball brought back? | |
| | | | Yes | 2 |
| | | | No | 1 |
| | | | Reluctant to let the ball go/ growls | 0 |
| 16 | Food bowl subtraction | Dog on lead, provide dog with full bowl of dry food, standing back. Dog approaches food, few mouthfuls allowed. Walk towards dog, use artificial hand to slightly pull bowl away. | Keeps eating ignoring tester | 3 |
| | | | Dog looks anxious and eats faster or moves away | 2 |
| | | | Dog is not interest in the food | 1 |
| | | | Dog growls at tester | 0 |
| 17 | Approaching large and small test dog | One large and one small tester dog is selected from each shelter. The tester dog is kept at a distance (2–3m). Both dogs | Attentive, confident and friendly | 2 |
| | | | Uninterested in other dog or | 1 |

| | | | | |
|---|---|---|---|---|
| | | are kept on their leads, and not to be in contact. | fearful | |
| | | | Stiff dominant posture/ growling | 0 |
| 18 | Approaching large and small artificial dog | Same procedure as subtest using fake dogs | Attentive, confident and friendly | 2 |
| | | | Uninterested in other dog or fearful | 1 |
| | | | Stiff dominant posture/ growling | 0 |
| 19 | Reactivity | a. Standing roughly 1m away from dog, making no eye contact, using a device that makes a loud noise, records dogs' behaviour. | Interested and explores stimuli | 3 |
| | | | Shows no reaction | 2 |
| | | | Fearful/ retreats | 1 |
| | | | Aggressive reaction | 0 |
| | | Repetition for habituation (10 trails, then cease if dog appears to be stressed) | Presence of habituation to stimuli: | |
| | | | Yes | 1 |
| | | | No | 0 |
| | | b. Standing roughly 1m away from dog, opening an umbrella in a repeating action | Interested and explores stimuli | 3 |
| | | | Shows no reaction | 2 |
| | | | Fearful/ retreats | 1 |
| | | | Aggressive reaction | 0 |
| | | Repetition for habituation (10 trails, then cease if dog appears to be stressed) | Presence of habituation to stimuli: | |
| | | | Yes | 1 |
| | | | No | 0 |
| 20 | Return to kennel | Return to original location on lead | Enters kennel immediately | 2 |
| | | | Reluctant to enter | 1 |
| | | | Pulls strongly/ refuses | 0 |

**Supplementary Table S2.** Ethogram used to score the behaviour of the tested dogs when presented with either a real or a fake dog opponent. Behaviours were scored in term of duration (**d**) of frequency (**f**).

| Behaviour | Description |
|---|---|
| Barking (f) | Loud, staccato vocalization |
| Deflection (d) | The dog obviously looks away (breaks eye contact) from the stimulus, or positions its body facing away from the stimulus |
| Direct staring/stiff posture (d) | The dog is staring at the stimulus. Often the pupils are slightly widened, ears up and forward, muscles in the body are tense, the dog looks stiff. Hairs on neck, back and hindquarters rise, tail is stiff and held upwards anywhere between a horizontal and vertical position |
| Growling/baring teeth/pulling lips up (d) | Dog produces low buzzing sound, may pull up its upper lip, so that its teeth are more or less visible |
| Investigate environment (d) | Dog explores visually or smells environment (excluding stimulus) e.g. floor, furniture etc. |
| Investigate stimulus (d) | Look at the stimulus in attentive manner and/or sniffing at the stimulus |
| Jump on handler (f) | Dog climb on handler legs or jumps bouncing off the handler |
| Jumping (f) | Dog jumping up and around the front of the handler, could be more or less pulling toward stimulus |
| Lie down (d) | Lie down on the floor |
| Lip/nose licking (f) | The dog's tongue appears from the front of the mouth and licks the upper lip or nostril |
| Look handler (d) | Dog looks at the handler, normally disengaging contact from the stimulus |
| Paw lift (d) | Dog lifts front paw, static or motions towards stimulus |
| Play bow (d) | Dog brings his front legs out in front of him and his chest low to the ground, his rear end remains up |
| Pull towards (d) | Dog pulls clearly toward the stimulus (no jumping) either in a social manner or confident/threatening one. |

| | |
|---|---|
| Pulling away (d) | Pulling in opposite direction to that of the stimulus |
| Shake off (f) | Oscillate vigorously the head and body on its longitudinal axis |
| Shrinking back (d) | The dog shrinks backward or sideward increasing the distance from the stimulus, this can include positioning itself behind the handler |
| Snapping/lounging (f) | Lunge forward, may be accompanied by aggressive barking or a snapping movement (mouth opens and closes) or a quick head movement. |
| Very low posture/tucked tail (d) | cowering down and trying to hide with respect to the stimulus, maybe freezing or trembling, tail tucked very low between legs |
| Wagging tail (d) | Dog is interested in the stimulus, looks at stimulus with tail wags forcefully from side to side or moves in a circular pattern, could be still or walking toward stimulus |
| Yawn (f) | Action of yawning |

**Supplementary Table S3.** Statistical test and p-values used to investigate whether the PC scores from the dog-dog interaction test differed in relation to the stimulus dogs' size (small/large) and nature (real/fake).

| Comparison | Confidence | Cautiousness | Assertiveness | Fearfulness |
|---|---|---|---|---|
| Fake vs Real | | | | |
| Wilcoxon W | 7385.000 | 7278.000 | 7358.000 | 7142.000 |
| *p*-value | 0.869 | 0.622 | 0.804 | 0.363 |
| Small vs Large | | | | |
| Wilcoxon W | 7388.000 | 7182.000 | 7056.000 | 7232.000 |
| *p*-value | 0.876 | 0.431 | 0.241 | 0.526 |

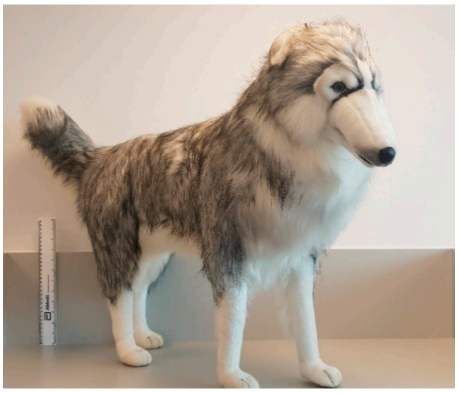

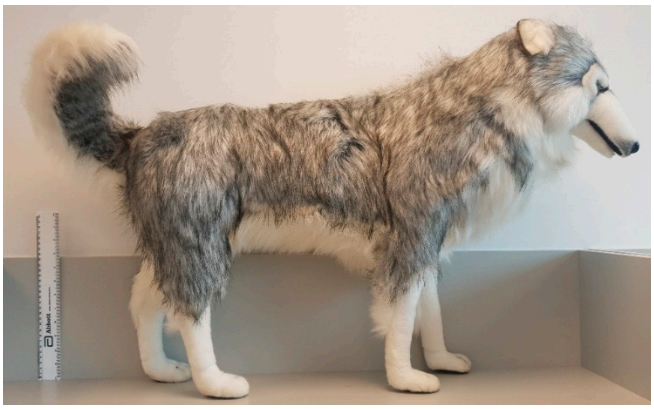

(**a**) (**b**)

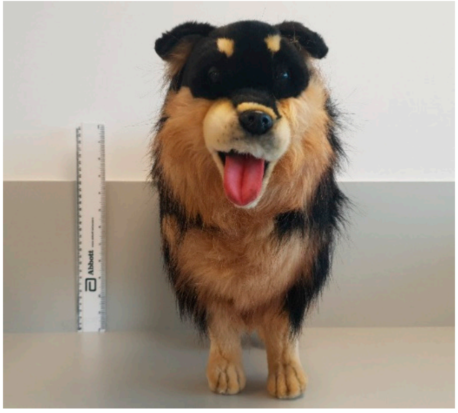

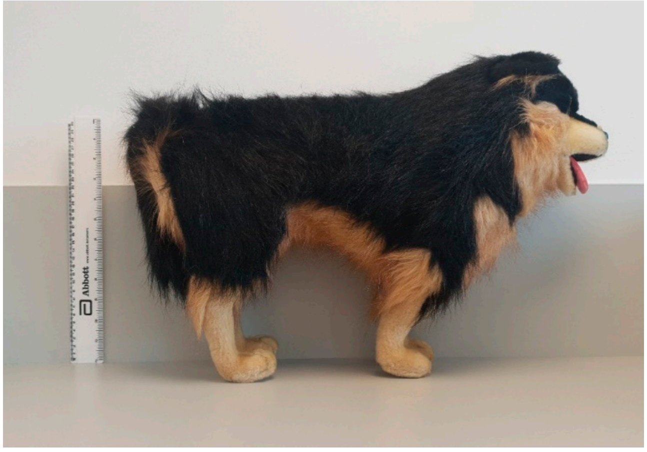

(**c**) (**d**)

**Supplementary Figure S1**: Large (**a**, **b**) and small (**c**, **d**) fake dogs used for testing.

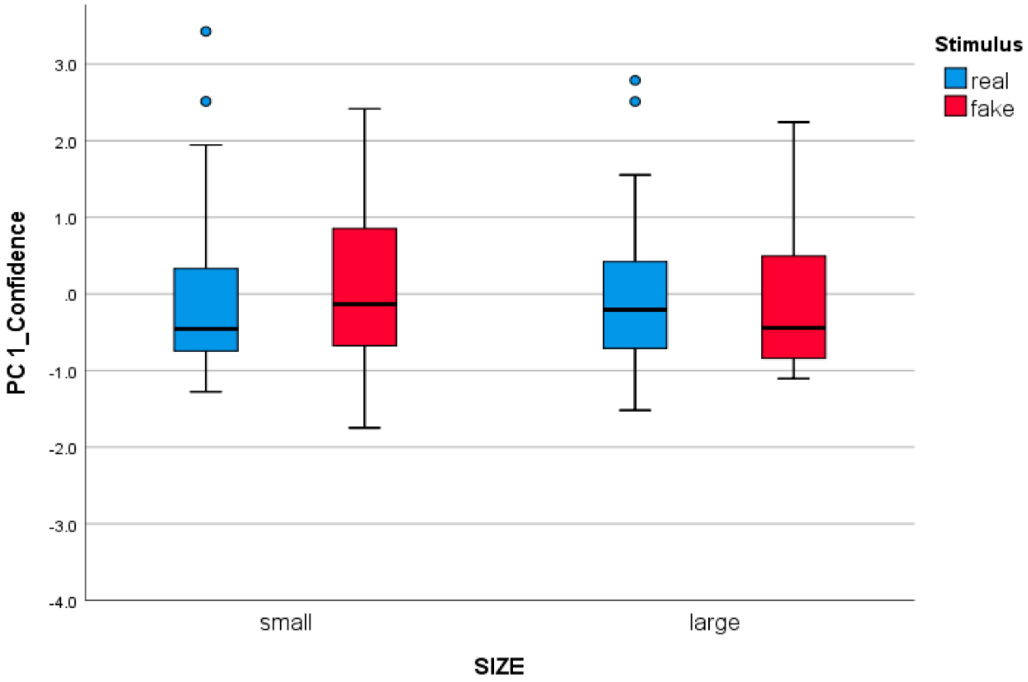


(**a**)

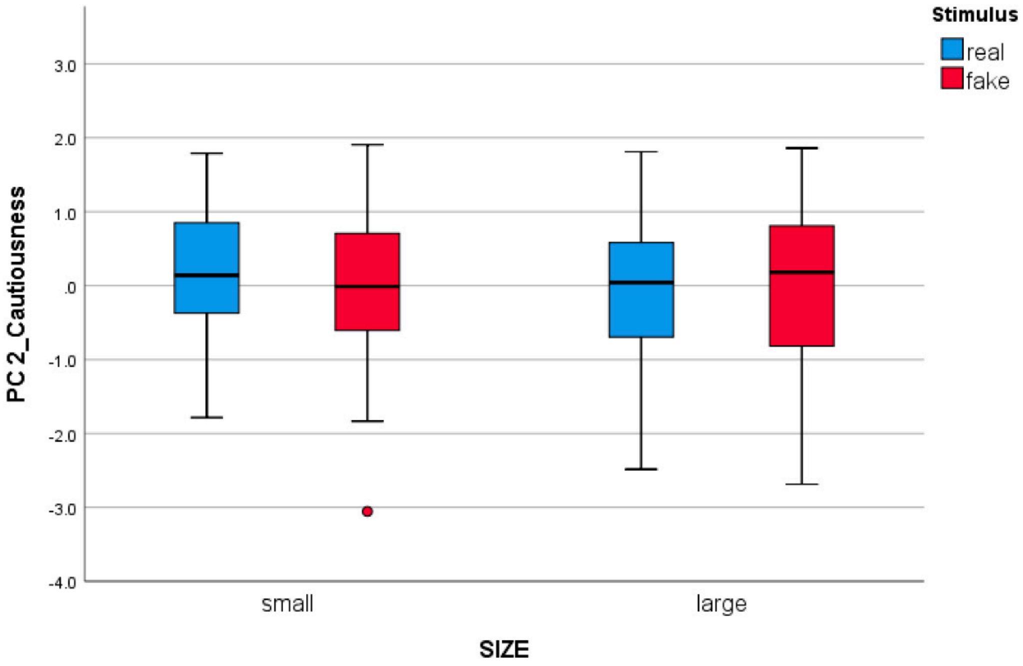
Stimulus
real
fake
PC 2_Cautiousness
3.0
2.0
1.0
.0
-1.0
-2.0
-3.0
-4.0
small
large
SIZE

(**b**)

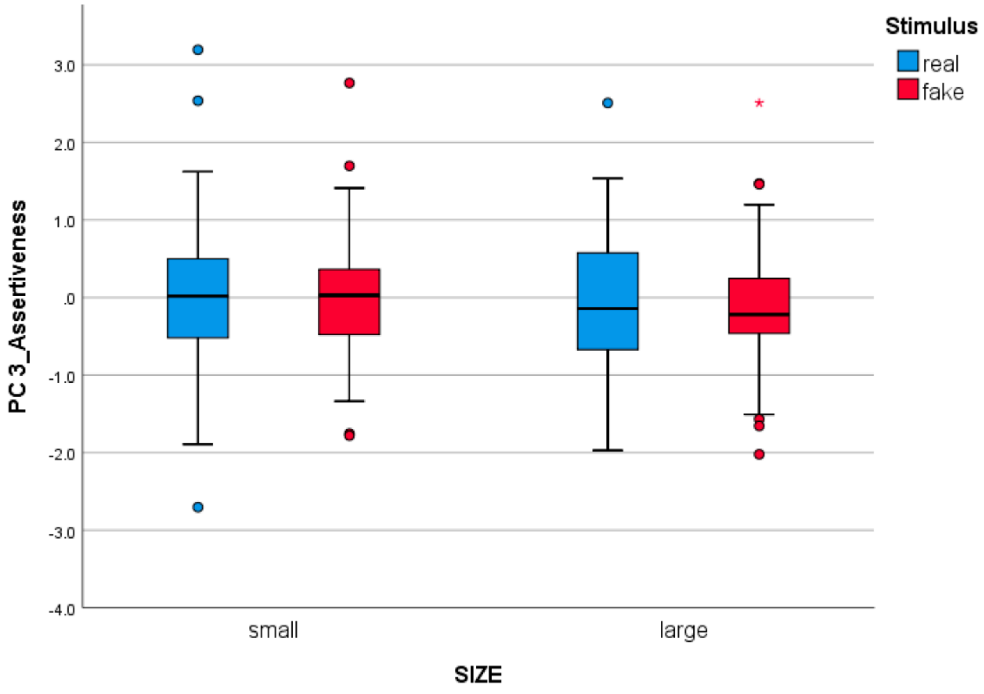
Stimulus
real
fake
PC 3_Assertiveness
3.0
2.0
1.0
.0
-1.0
-2.0
-3.0
-4.0
small
large
SIZE

(**c**)

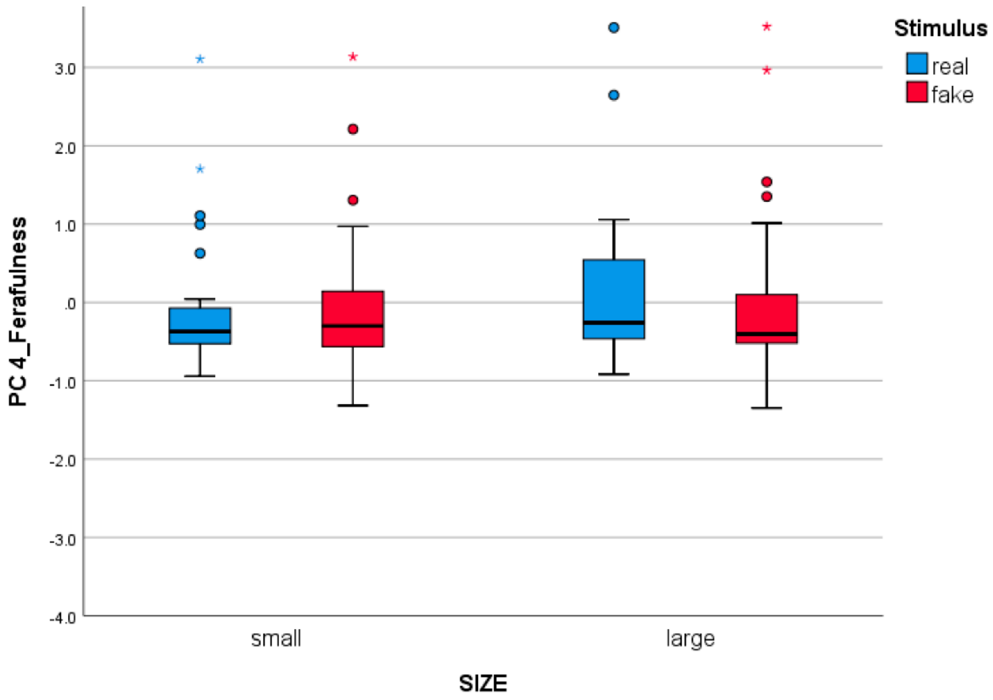


(**d**)

**Supplementary Figure S2.** Boxplot showing group comparisons for (**a**) the first PCA component scores 'confidence' and (**b**) the second PCA component scores 'cautiousness' (**c**) the third component scores 'assertiveness' and (**d**) the final component score 'fearfulness' in response to the different stimulus dogs' characteristics (real vs fake and small vs large). Dots represent outliers.
